# Redundant G_s_-coupled serotonin receptors regulate amyloid-β metabolism in vivo

**DOI:** 10.1186/s13024-016-0112-5

**Published:** 2016-06-18

**Authors:** Jonathan R. Fisher, Clare E. Wallace, Danielle L. Tripoli, Yvette I. Sheline, John R. Cirrito

**Affiliations:** Department of Neurology, Washington University School of Medicine, St. Louis, MO USA; Knight Alzheimer’s Disease Research Center, Washington University School of Medicine, St. Louis, MO USA; Hope Center for Neurological Disorders, Washington University School of Medicine, St. Louis, MO USA; Departments of Psychiatry, Radiology, and Neurology, University of Pennsylvania, Philadelphia, PA USA; Present Address: Washington University, Neurology, 660 South Euclid Avenue, Campus Box 8111, St. Louis, MO 63110 USA

**Keywords:** Alzheimer’s Disease, beta-amyloid, Serotonin receptor, SSRI, Microdialysis, PKA, α-secretase

## Abstract

**Background:**

The aggregation of amyloid-β (Aβ) into insoluble plaques is a hallmark pathology of Alzheimer’s disease (AD). Previous work has shown increasing serotonin levels with selective serotonin re-uptake inhibitor (SSRI) compounds reduces Aβ in the brain interstitial fluid (ISF) in a mouse model of AD and in the cerebrospinal fluid of humans. We investigated which serotonin receptor (5-HTR) subtypes and downstream effectors were responsible for this reduction.

**Results:**

Agonists of 5-HT_4_R, 5-HT_6_R, and 5-HT_7_R significantly reduced ISF Aβ, but agonists of other receptor subtypes did not. Additionally, inhibition of Protein Kinase A (PKA) blocked the effects of citalopram, an SSRI, on ISF Aβ levels. Serotonin signaling does not appear to change gene expression to reduce Aβ levels in acute timeframes, but likely acts within the cytoplasm to increase α-secretase enzymatic activity. Broad pharmacological inhibition of putative α-secretases increased ISF Aβ and blocked the effects of citalopram.

**Conclusions:**

In total, these studies map the major signaling components linking serotonin receptors to suppression of brain ISF Aβ. These results suggest the reduction in ISF Aβ is mediated by a select group of 5-HTRs and open future avenues for targeted therapy of AD.

**Electronic supplementary material:**

The online version of this article (doi:10.1186/s13024-016-0112-5) contains supplementary material, which is available to authorized users.

## Background

One primary pathology of Alzheimer’s disease (AD) is the accumulation of insoluble amyloid-β (Aβ) plaques in the brain. Aβ is produced by sequential cleavage of the amyloid precursor protein (APP) by β- and γ-secretases [1, 2]. Alternatively, activity of another type of enzymes, α-secretases, can cleave within the Aβ sequence and prevent its formation [3]. Concentration is a key factor that determines whether soluble Aβ peptide aggregates into oligomers and plaques [4, 5], with higher concentrations increasing the likelihood that toxic multimers of Aβ will form. Consequently, reducing Aβ levels is one promising target for AD therapy.

Serotonin, Aβ, and AD have been connected in several studies in the scientific literature. The amount of both serotonin (5-HT) and its receptors (5-HTRs) are reduced in human post-mortem AD studies [6, 7]. Serotonergic activity reduces Aβ production rates in vitro and 5-HTR agonists alter APP processing to increase soluble fragments of the protein that are consistent with a suppression in Aβ generation [8–11]. Manipulating serotonin levels in vivo shows similar effects. In our previous work, treating *APPswe/PS1∆E9* (APP/PS1) mice with a single dose of a selective serotonin re-uptake inhibitor (SSRI) reduced brain interstitial fluid (ISF) Aβ concentrations by 25 % [12]. Serotonin treatment did not alter the Aβ elimination rate which suggests this reduction was not mediated by Aβ clearance mechanisms. Instead, α-secretase enzymatic activity was increased by SSRI treatment, suggesting that Aβ generation was suppressed. Chronic dosing with a SSRI over 4 months reduced brain Aβ plaque load and cerebrospinal fluid (CSF) Aβ levels in mice by 50 % [12]. Similar reductions in Aβ were seen by SSRI treatment of 3xTg AD mouse model [6]. The reduction in Aβ by SSRI is not limited to mice; young adult, cognitively normal, non-depressed individuals given a single dose of the SSRI citalopram also showed a reduction in CSF Aβ levels in a matter of hours [13].

The cellular mechanism of SSRI-induced Aβ reduction is likely complex. There are 15 identified serotonin receptors expressed in the brain [14]. Most 5-HTRs are G-protein coupled receptors (GPCRs) while 5-HT_3_R is the only ionotropic cation channel [15]. 5-HT_1_R and 5-HT_5_R couple to G_i/o_ signal proteins and typically lead to Protein Kinase C (PKC) activation [16–18] while 5-HT_2_R signals through G_q_ proteins to activate calcium-calmodulin dependent kinase II (CaMKII) [15, 19]. 5-HT_4_R, 5-HT_6_R, and 5-HT_7_R activate G_s_ proteins, which usually increase cyclic AMP levels, and induce Protein Kinase A (PKA) activation [10, 15, 20]. Interestingly, stimulating some serotonin receptors can activate the extracellular signal-regulated kinase (ERK) [18, 20–22]. Inhibition of MAP kinase-ERK kinase (MEK), the kinase that activates ERK, reduces the production of the α-secretase cleavage product sAPPα in vitro [23]. Also, inhibiting either ERK or MEK increased ISF Aβ levels in mice and blocked the SSRI-dependent reduction in Aβ [12]. Our current study shows serotonin-induced reductions of Aβ, rely on the G_s_-linked serotonin receptors and PKA signaling. We also provide evidence that serotonergic-dependent suppression of Aβ is mediated through an increase in α-secretase enzymatic activity.

## Results

### Serotonin-induced reduction in ISF Aβ is receptor specific

Aβ is largely produced by neurons during synaptic activity and is continually released into the ISF [24–27]. We used in vivo brain microdialysis to measure dynamic changes in ISF Aβ levels [28]. This method allows for serial collection of ISF Aβ every hour over the course of several days from awake and freely mobile mice [24]. Small molecule compounds can be administered by intraperitoneal (i.p.) injection or by infusing the agents directly into the brain via the microdialysis probe, a method called reverse microdialysis when agents cross the membrane and act locally around the probe. Local infusion circumvents the blood brain barrier and allows for continual administration over time.

**Fig. 1:**
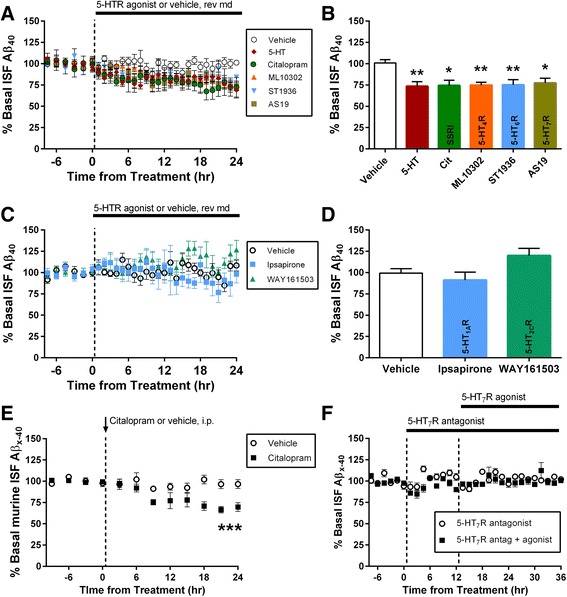
A small subset of 5-HT receptors reduce ISF Aβ levels in vivo. Selective agonists for individual 5-HT receptors or vehicle (DMSO) were infused via reverse microdialysis (rev md) in 2–3 month old APP/PS1 hemizygous mice. **a** As assessed by microdialysis, broad activation of 5-HTRs by serotonin (2 mM by rev md) directly or by citalopram (10 mg/kg i.p.), a SSRI, caused a decrease in ISF Aβ levels. Similarly, reverse microdialysis treatment with agonists for 5-HT_4_R (ML10302, 400 nM), 5-HT_6_R (ST1936, 1.3 μM), and 5-HT_7_R (AS19, 83 nM) induced a reduction in ISF Aβ in living mice. **b** After hours 22-24 of continuous treatment, ML10302, ST1936, and AS19 reduced ISF Aβ_x-40_ by 25.2 ± 3.4 % (p < 0.01; *n* = 6), by 24.8 ± 6.1 % (*p* < 0.01; *n* = 6), and by 22.5 ± 5.5 % (*p* < 0.05; *n* = 6), respectively. Serotonin and citalopram also significantly reduced ISF Aβ by 26.5 ± 5.4 % (*p* < 0.01; *n* = 7) and 25.5 ± 3.4 % (*p* < 0.05; *n* = 5), respectively. **c** Agonist treatment for 5-HT_1A_R (Ipsapirone, 1 μM) or 5-HT_2C_R (WAY161503, 400 nM) showed no significant reduction of ISF Aβ. **d** After 24 h of continuous treatment, Ipsapirone reduced ISF Aβ_x-40_ to 91.2 ± 9.4 % (*n* = 6) and WAY161503 increased ISF Aβ_x-40_ to 119.9 ± 8.6 % (*n* = 8). **e** Citalopram (10 mg/kg, i.p.) administered to young C57Bl6 wildtype mice significantly reduced ISF Aβ levels by 32 ± 4.2 % at 21–24 h after treatment compared to vehicle-treated (PBS) mice (*p* < 0.001; *n* = 6). **f** Mice were treated with selective 5-HT_7_R antagonist SB258719 (3.16 μM) for 8 h followed by co-administration of 5-HT_7_R agonist, AS19, (83nM) for 24 h by reverse microdialysis (*n* = 4). The antagonist completely blocked the effect of the agonist. The antagonist alone had no significant effect on ISF Aβ levels. Data presented as mean ± SEM. Asterisks mark *p-*values < 0.05; double asterisks mark *p-*values < 0.01; triple asterisks mark *p-*values 0.001

We previously showed that treating mice with several SSRIs caused a 25 % decrease in ISF Aβ [12]. SSRI compounds are not selective for a specific 5-HTR; they compete with 5-HT for binding to the serotonin reuptake transporter which increases 5-HT concentrations [29], thus activating all serotonin receptors present. The SSRI-dependent reduction in brain Aβ requires ERK activation, and stimulation of 5-HTRs can stimulate ERK under certain conditions [12, 18, 20–22]. These results suggest that any or all of the 15 different 5-HTRs could be responsible for the reduction in ISF Aβ. To determine the role of individual receptors, we administered a selective 5-HTR agonist for each receptor class by reverse microdialysis. Two to three month old APP/PS1 mice [30] were implanted with unilateral microdialysis probes in the hippocampus. Mice at this age do not yet show Aβ plaques which enabled us to study normal Aβ metabolism without the added variable of Aβ pathology [28, 31, 32]. Basal levels of ISF Aβ were evaluated for 9 h before treatment with selective 5-HTR agonists by reverse microdialysis (see Table 1 for dosages). Treatment with a selective agonist for 5-HT_4_R, 5-HT_6_R, or 5-HT_7_R significantly reduced ISF Aβ_40_ by approximately 25 %. Each of these three receptors was equally effective at lowering Aβ. Interestingly, reductions by each agonist alone are equivalent to reduction induced by 5-HT or citalopram SSRI (Fig. 1a and b), suggesting that each receptor alone is sufficient to induce the maximal effect on ISF Aβ. However, treatment with agonists selective for 5-HT_1A_R or 5-HT_2C_R did not produce significant changes in ISF Aβ_40_ (Fig. 1c and d). These results suggest that only a subset of 5-HTRs, specifically those that activate G_s_-coupled proteins, are likely responsible for the reduction in ISF Aβ by serotonin. To ensure that the serotonin-mediated suppression in ISF Aβ is via a normal metabolic pathway and not an artifact of APP transgenic over-expression, we administered citalopram to wildtype, C57Bl6 mice. The SSRI reduced ISF murine Aβ levels by over 30 % compared to vehicle, similar to the effect found in transgenic mice (Fig. 1e).

**Table 1:** Pharmacological agents used for reverse microdialysis

Compound	Target	IC_50_ or K_i_	Rev MD Concentration	Dosage Across MD Membrane	Selectivity Limit	Reference
Agonists						
Ipsapirone	5-HT_1A_R	10 nM	1 μM	100 nM	58 nM^a^	[59]
WAY161503	5-HT_2C_R	4 nM	400 nM	40 nM	233 nM	[60]
ML10302	5-HT_4_R	4 nM	400 nM	40 nM	700 nM	[61]
ST1936	5-HT_6_R	13 nM	1.3 μM	130 nM	168 nM	[62]
AS19	5-HT_7_R	0.83 nM	83 nM	8.3 nM	6.6 nM^b^	[63]
Antagonists						
GR113808	5-HT_4_R	0.02 nM	100 nM	10 nM	10 μM	[64]
SB258719	5-HT_7_R	31.6 nM	3.16 μM	316 nM	316 nM	[68]
Inhibitors						
GM6001	ADAM/MMP	0.5 nM-27 nM	2.5 μM	250 nM	NA	[66]
KT5720	PKA	60 nM	6 μM	600 nM	2 μM	[67]
PKI14-22	PKA	36 nM	3.6 μM	360 nM	15 μM	[65]

All agents were chosen for their selectivity for their targets as assayed in previous literature. We used the K_i_ and IC_50_ values to determine necessary concentrations of each compound (reverse microdialysis (Rev MD) concentration). We assumed only 10 % of the drug would cross the membrane (Dosage Across MD membrane), and this amount of the compound would be further diluted in the brain ISF. The selectivity limit is the concentration above which each compound begins stimulating off-target receptors

^a^Ipsapirone has been shown to stimulate bovine α-adrenergic receptors at 58 nM in vitro [59]

^b^AS19 can stimulate 5-HT_1D_R at 6.6 nM concentrations in vitro [63]. However, expression of this receptor is absent in adult mouse hippocampus [14], so effects of its stimulation should be neglible

Abbreviations: *5-HTR* serotonin receptor, *ADAM* a disintegrin and metallopeptidase domain, *MMP* matrix metallopeptidase, *PKA* protein kinase A

The dose of each agonist was 5-175 times more selective for their targets than other receptor types. We estimate that only 10 % of each compound crosses the microdialysis membrane; however, this varies based on drug properties so we cannot rule out some off-target effects of these compounds. The concentration of 5-HT_1A_R agonist we used in this experiment could possibly activate α-adrenergic receptors, though we saw no reduction in Aβ levels like those seen with the potent adrenergic receptor agonist caffeine [33]. To verify that the reductions in ISF Aβ were indeed to due to 5-HTR activation, we co-administered a 5-HT_7_R antagonist and agonist to APP/PS1 mice (Fig. 1f). Pretreatment with the antagonist completely blocked the reduction in ISF Aβ by the agonist, demonstrating the specificity of this suppression in ISF Aβ is due to those receptors.

Citalopram also significantly reduced soluble APPα (sAPPα) levels in the ISF of young APP/PS1 by 17.4 ± 4.0 % (p <0.05) as assessed with 1,000 kDa MWCO microdialysis probes and assessed by Western blotting (Additional file 1: Figure S1). However, the SSRI had no effect on ISF Aβ oligomers in these young mice (Additional file 1: Figure S2).

### Selective 5-HTR antagonists increase ISF Aβ

**Fig. 2:**
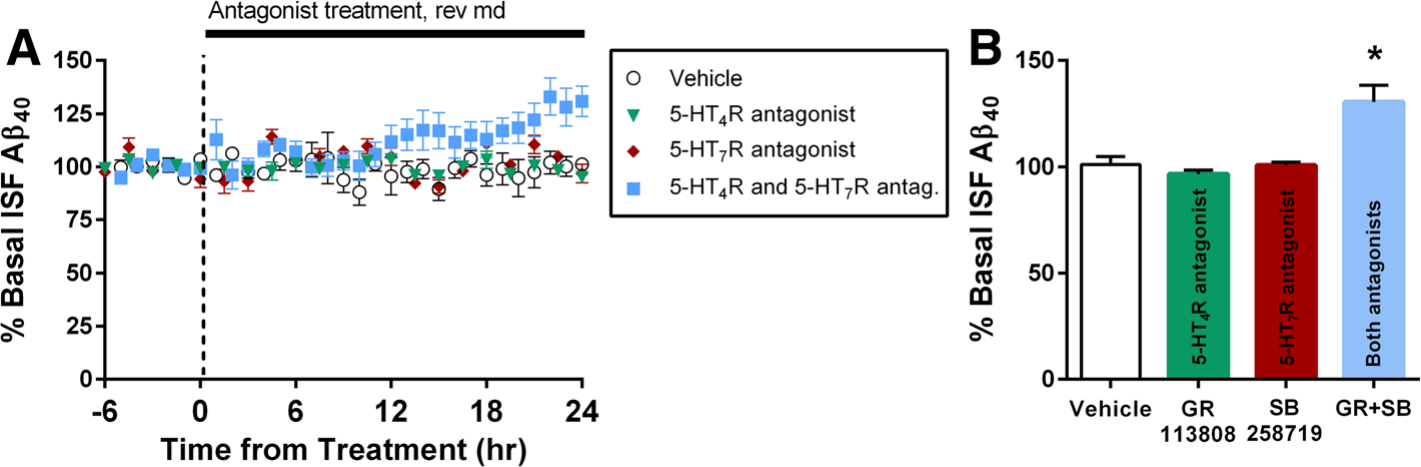
Inhibiting 5-HT_4_R and 5-HT_7_R simultaneously increases ISF Aβ. Selective 5-HT_4_R antagonist GR113808 (100 nM), selective 5-HT_7_R antagonist SB258719 (3.16 μM), both antagonists together, or DMSO vehicle were infused via reverse microdialysis in 2.5 month old APP/PS1 hemizygous mice for 8 h. **a** As assayed by microdialysis, treatment with GR113808 or SB258719 alone had no detectable effect on ISF Aβ_x-40_ (*n* = 4), but co-administration of both antagonists increased ISF Aβ_x-40_. **b** 24 h of treatment with both antagonists significantly increased ISF Aβ_x-40_ to 132 ± 11.2 % (*p* = 0.048, *n* = 10) by 21–24 h. Data presented as mean ± SEM. Asterisks mark *p-*values < 0.05

If 5-HT_4_R, 5-HT_6_R, and 5-HT_7_R mediated the reduction in ISF Aβ, then we postulated that blocking their activity may have the opposite effect. To test this hypothesis, we administered GR113808 and SB258719, selective antagonists for 5-HT_4_R and 5-HT_7_R respectively, alone or in combination via reverse microdialysis (Fig. 2a). We chose to examine 5-HT_4_R and 5-HT_7_R as there is evidence their expression is reduced with increasing age [34]. Antagonizing a single receptor did not significantly change Aβ_40_ levels after 24 h of administration. However, blocking both receptors caused a significant increase of 32 % in ISF Aβ_40_ (Fig. 2b). Taken together with data from Fig. 1a, our results demonstrate that basal activity of 5-HT_4_R and 5-HT_7_R is normally suppressing Aβ levels, but stimulated activity of only one receptor at a time is sufficient to reduce Aβ levels in acute time frames. This suggests there is a redundancy to 5-HT signaling that is capable of lowering Aβ levels.

### Inhibition of PKA activity increases ISF Aβ levels

**Fig. 3:**
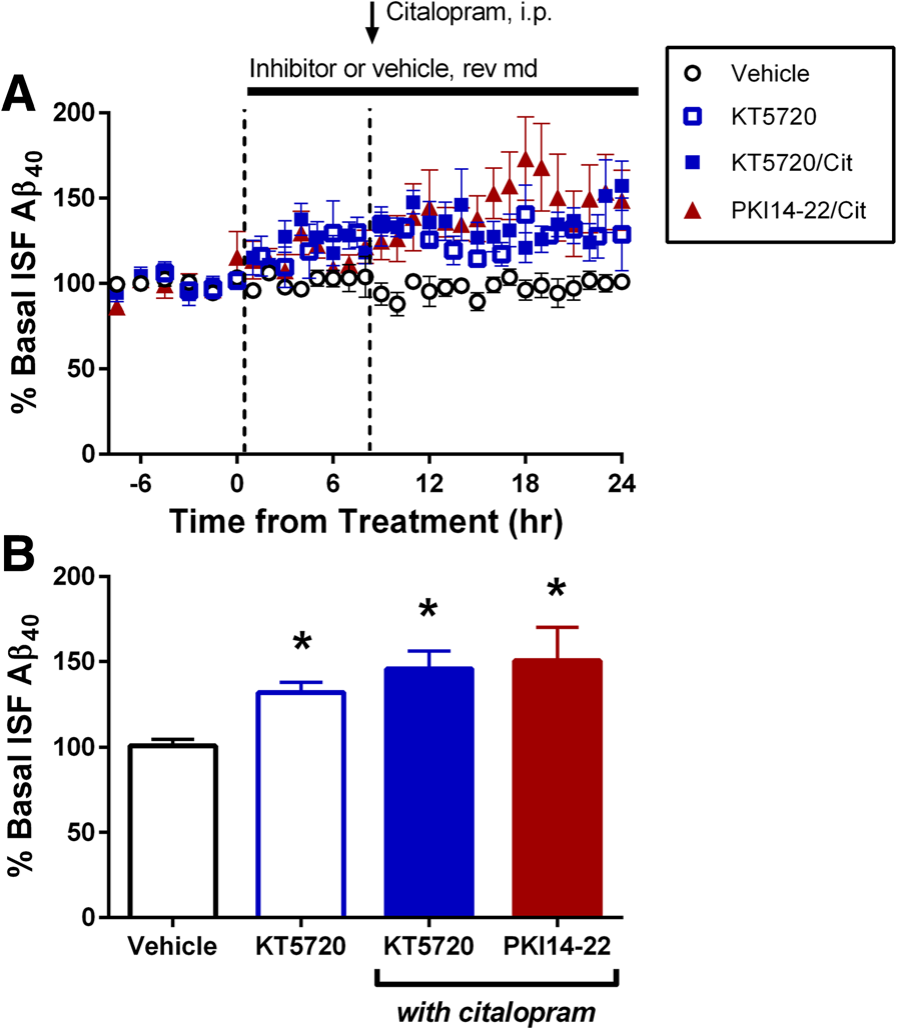
PKA activity modulates production of ISF Aβ. **a** Selective small molecule inhibitor KT5720 (6 μM), peptide inhibitor PKI14-22 amide (3.6 μM), or vehicle (DMSO or acetonitrile, respectively) were infused via reverse microdialysis (rev md) in 2.5 month old APP/PS1 hemizygous mice for 24 h. In two cohorts of mice after 8 h of inhibitor treatment, the SSRI citalopram was administered by i.p. injection at 10 mg/kg. **b** Inhibiting PKA with KT5720 alone for 24 h caused a significant increase in ISF Aβ_x-40_ levels by 31.9 ± 6.0 % (*p* < 0.05; *n* = 4). By 16 h after co-administration of citalopram with either KT5720 or PKI 14-22, ISF Aβ_x-40_ levels were significantly increased by 45.8 ± 10.5 % (*p* < 0.05, *n* = 6) and by 50.6 ± 19.6 % (*p* < 0.05, *n* = 6), respectively. Most importantly, each inhibitor completely blocked the effect of citalopram on ISF Aβ levels. Data represented as mean ± SEM. Asterisks mark *p*-values < 0.05

The strong effects of 5-HT_4_R, 5-HT_6_R, and 5-HT_7_R led us to examine downstream signaling pathways. All three of these receptors typically activate G_s_ proteins, increase cAMP levels, and induce PKA activation [15, 20]. To determine the role of PKA in serotonin-mediated ISF Aβ reduction, we administered KT5720, a small molecule selective PKA inhibitor, via reverse microdialysis. Treatment with KT5720 alone for 24 h significantly increased ISF Aβ levels by 30 %, suggesting PKA has a normal role in regulating basal ISF Aβ levels (Fig. 3a and b). Our previous results suggest that 5-HT_4_R, 5-HT_6_R, and 5-HT_7_R work together to regulate basal ISF Aβ levels; therefore, we stimulated all receptors with a SSRI in order to observe the effects of PKA inhibition. The KT5720 inhibitor also blocked the citalopram-dependent decrease in ISF Aβ levels. To confirm the specificity of the results, we replicated the experiment with a structurally-distinct PKA peptide inhibitor, PKI14-22 amide myristoylate [35]. PKI14-22 was pretreated by reverse microdialysis for 8 h, followed by co-administration of citalopram by i.p. injection (10 mg/kg). Both inhibitors of PKA completely blocked the effect of the SSRI (Fig. 3b). These results strongly suggest PKA activity is required for ISF Aβ reduction by 5-HT signaling.

### Acute SSRI treatment does not change expression of Aβ metabolism-related genes/proteins

**Fig. 4:**
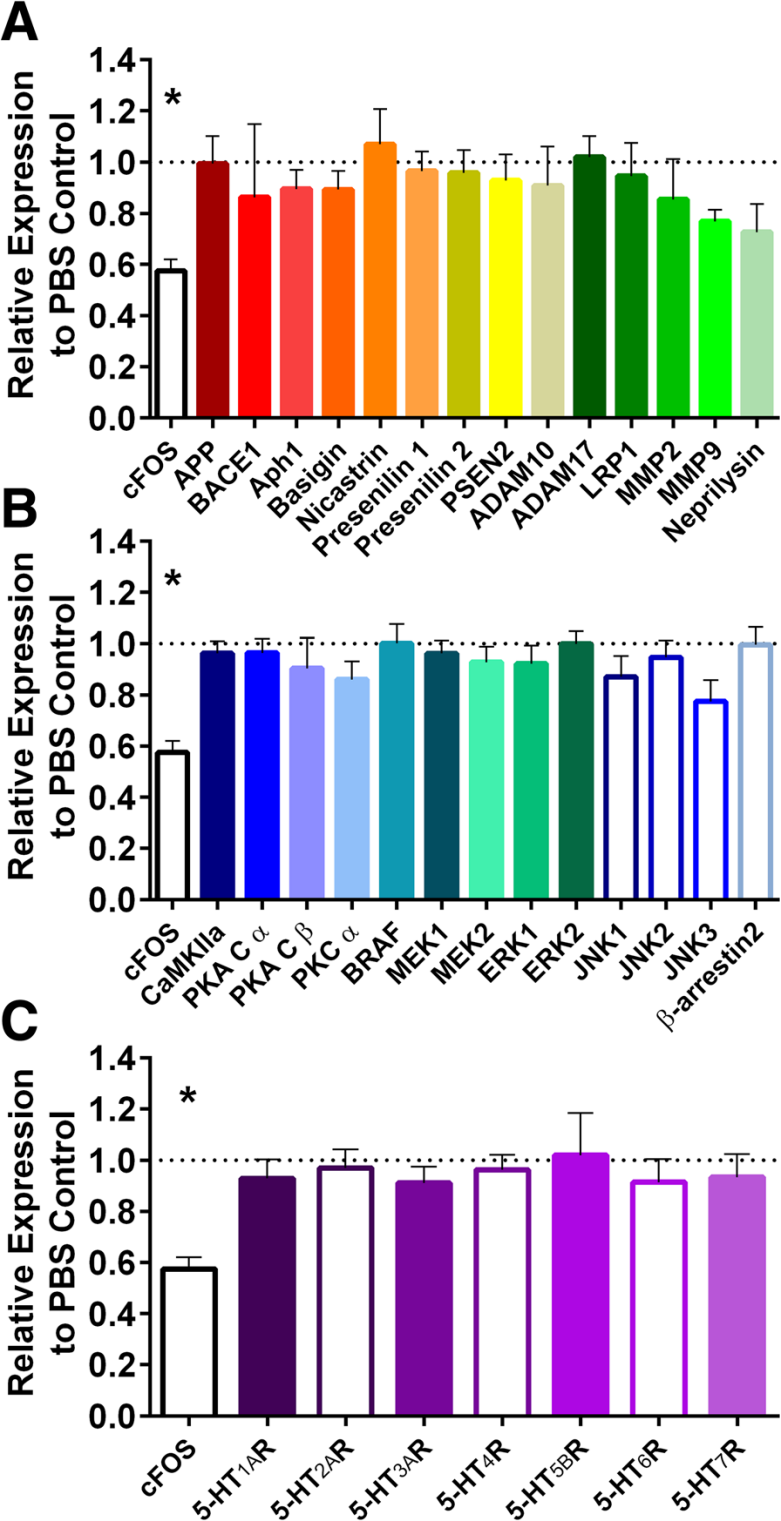
Acute SSRI treatment has no significant effect on expression of Aβ APP metabolism genes. 2.5 month old APP/PS1 hemizygous mice were treated with 10 mg/kg citalopram or PBS by i.p. injection. 16 h later hippocampi were microdissected for qPCR analysis. **a** qPCR analysis for genes involved in Aβ processing or clearance showed no significant changes in expression. In contrast, the positive control, cFos*,* was significantly reduced to 57.5 ± 0.04 % (*p* < 0.05). **b** qPCR analysis for genes encoding signaling proteins downstream of 5-HTR showed no significant changes in expression. **c** qPCR analysis for 5-HTR genes showed no significant changes in expression. Data are presented as mean ± SEM. Values are normalized to the mean expression level of the PBS controls (*n* = 6). Statistical significance was calculated by one-way ANOVA with comparison to CaMKII and corrected for multiple comparisons. Asterisks mark *p-*values < 0.05. See Additional file 1 for primer sequences and database numbers

Activation of ERK is one event that can occur downstream of PKA signaling [15]. We have previously shown that pERK levels were elevated after SSRI treatment, and inhibiting the ERK signaling cascade increased ISF Aβ [12]. Once phosphorylated, activated ERK can trans-locate to the nucleus to modify nuclear proteins and suppress gene expression. Interestingly, reductions in secretase gene expression have been observed with chronic SSRI therapy in other studies [12, 36]. To determine if ERK is modifying gene expression to reduce ISF Aβ in the acute phase, we measured expression of genes involved in Aβ metabolism following treatment with citalopram. 2.5 month old APP/PS1 mice were treated with citalopram by i.p. injection (10 mg/kg) or PBS and were sacrificed 16 h later. This dosage of SSRI was enough to reduce ISF Aβ levels (Fig. 1), and 16 h was the earliest time that reductions in ISF Aβ reached statistical significance after SSRI treatment [12]. We performed quantitative real-time PCR (qPCR) for 34 genes involved in Aβ metabolism using purified hippocampal mRNA. Expression of immediate early gene FBJ osteosarcoma oncogene (cFOS) was used as a positive control; its expression is reduced by citalopram within 24 h [37]. Expression of each gene was normalized to glyceraldehyde 3-phosphate dehydrogenase (GAPDH) and normalized to the mean expression level of PBS-treated controls. CaMKII was used as the control for statistical analysis since our previous studies showed that inhibition of its activity had no effect on ISF Aβ levels [38], and we observed no changes in gene expression by qPCR. There was no significant change in APP expression following SSRI treatment. α-secretase (a disintegrin and metallopeptidase domain 10 (ADAM10), ADAM17, matrix metallopeptidase 9 (MMP9)), β-secretase (beta-site APP-cleaving enzyme (BACE1)), and γ-secretase (anterior pharynx defective 1 (Aph1), basigin, nicastrin, presenilin 1, presenilin 2, presenilin enhancer 2 (PSEN2)) gene expression were not significantly affected by SSRI. There was also no altered expression of MMP9, neprilysin, or low density lipoprotein receptor-related protein 1 (LRP1), three proteins involved with Aβ clearance (Fig. 4a). There was no significant change in major signaling genes such as PKA (PKA catalytic unit α (PKA Cα), PKA catalytic unit β (PKA Cβ), PKC (PKCα), CaMKII, MEK, ERK, or c-Jun N-terminal kinase (JNK). Expression of β-arrestin 2, an important scaffolding protein for many signaling pathways, showed no change after SSRI treatment. Expression of serotonin receptors was also not significantly changed following SSRI treatment (Fig. 4b and c). These negative results suggest that the acute reductions in ISF Aβ following citalopram treatment are unlikely to be mediated by changes in expression of genes linked to Aβ metabolism.

We also performed Western blots for several putative α-secretase proteins (ADAM10, ADAM17, MMP2) in hippocampi from mice treated with citalopram for 16 h (Additional file 1: Figure S3). We did not find any change in expression levels for these proteins in whole tissue extracts; however, we cannot discount the possibility of altered sub-cellular localization of these proteins or that their enzymatic activity could be enhanced [12] despite no change in expression.

### α-secretase mediates the serotonin-induced reduction in ISF Aβ

**Fig. 5:**
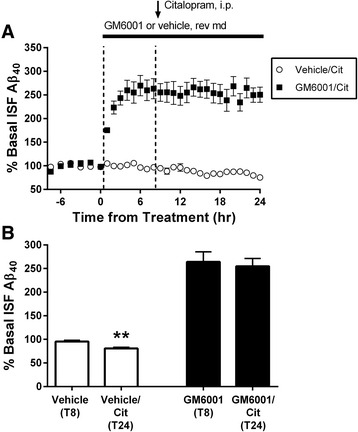
Broad spectrum inhibition of α-secretase enzymes blocks the effects of SSRI. Broad spectrum ADAM/MMP inhibitor GM6001 (25 μM) or vehicle (DMSO) was infused via reverse microdialysis in 2.5 month old APP/PS1 hemizygous mice. 8 h later, the SSRI citalopram was administered by i.p. injection at 10 mg/kg. **a** As assessed by microdialysis, inhibiting ADAM/MMP enzymes dramatically increased ISF Aβ_x-40_ levels, but there was no reduction in ISF Aβ after SSRI. **b** After 16 h, vehicle control with SSRI significantly reduced ISF Aβ_x-40_ to 80.9 ± 2.4 % (*p* = 0.002, *n* = 6) of levels at hour 8. GM6001 treatment increased ISF Aβ_x-40_ to 264.2 ± 21.1 % at time point 8. After 16 h of SSRI treatment, GM6001 ISF Aβ_x-40_ levels were unchanged at 254.9 ± 16.3 % of baseline (*p* = 0.74, *n* = 6). Data represented as mean ± SEM. Asterisks mark *p-*values < 0.05

We previously showed that α-secretase enzymatic activity was increased by SSRI while β-secretase activity was unchanged [12]. Also there was no difference in Aβ half-life after SSRI treatment which suggests that Aβ clearance was unaffected [12]. These results suggest the reduction of ISF Aβ by serotonin is induced by reducing Aβ production. Similar to other neurotransmitter receptors, we hypothesized that increased α-secretase activity may mediate this suppression of Aβ [38–40]. Several proteins demonstrate α-secretase enzymatic activity in vitro such as ADAM10, ADAM17, and MMP9 [3, 41, 42]. The presence of multiple α-secretase enzymes can generate redundancy; therefore, inhibiting a single candidate may not stop all enzyme activity [43, 44]. Consequently, we administered the broad spectrum ADAM/MMP inhibitor, GM6001, by reverse microdialysis for 8 h and then co-administered citalopram by i.p. injection. Treatment with 25 μM GM6001 induced a dramatic increase in ISF Aβ, but levels stabilized near 260 % of baseline after a few hours (Fig. 5a). However, ISF Aβ levels for GM6001-treated mice were not significantly reduced after 16 h of citalopram treatment (Fig. 5b), suggesting that serotonin-induced reduction in ISF Aβ is mediated by α-secretase enzymatic activity.

## Discussion

We found that only a subset of 5-HTRs are required to suppress brain Aβ levels in vivo. Serotonin receptors that activate PKA subsequently activate ERK to increase α-secretase enzymatic activity. This increased activity cleaves APP within the Aβ sequence to suppress brain ISF Aβ levels.

### ISF Aβ levels are reduced by specific 5-HTRs signaling through PKA

Stimulation of serotonin receptors with SSRI antidepressants reduces brain ISF Aβ by 25 % in APP/PS1 transgenic mice [12]. As SSRI compounds are not selective for a specific 5-HTR, therefore any or all of the receptors could reduce ISF Aβ. In this study we demonstrate that stimulation of 5-HT_4_R, 5-HT_6_R, and 5-HT_7_R with selective agonists significantly lowered ISF Aβ while agonists for other 5-HTR subtypes did not (Fig. 1). Interestingly, the magnitude of reduction by 5-HT_4_R, 5-HT_6_R, or 5-HT_7_R activity alone was equal to the reduction induced by serotonin or SSRI. Further, blocking activity of one receptor has no effect on Aβ; blocking two of these receptors together is necessary to increase ISF Aβ (Figs. 1 and 2). This suggests there is a redundancy and compensation in the serotonin signaling pathway that is capable of suppressing Aβ generation. These results suggest that activity of only three 5-HTRs are primarily responsible for the reduction in Aβ after SSRI treatment. Our data are consistent with previous findings in the literature. Tesseur et al. showed 1 month treatment with a 5-HT_4_R selective agonist reduced soluble Aβ_40_ and plaque load in the hippocampus [45]. Also, Gianonni et al. showed therapy with a weak 5-HT_4_R agonist increased concentrations of the α-secretase product and reduced plaque load in 5xFAD mice [46]. 5-HTR subtypes 4, 6 and 7 are G_s_-linked receptors that typically activate PKA. Tesseur et al. reported that 5-HT_4_Rs activate phospholipase C (PLC) to reduce Aβ levels [45]; further studies are necessary to determine if PKA and PLC work in concert or are mutually-exclusive pathways to modulate Aβ levels. It is worth noting that we used agonists for 5-HT 1A, 2C, 4, 6, and 7 receptors as representatives for each class. However, we cannot draw absolute conclusions whether other 5-HTR subtypes may have similar or even contrary effects on Aβ levels. Additionally, activation of 5-HT_2C_R had a trend to increase ISF Aβ levels (*p* = 0.0625), suggesting that other receptor subtypes may have the opposite effect on ISF Aβ. Whether or not other 5-HT receptors have opposing effects on Aβ remains to be explored. It is important to note the limitation that few compounds tested had a full dose–response performed to determine their most effective dose in vivo. Most of these selective compounds begin stimulating off-target receptors above a specific concentration. For example, a higher dosage for the 5-HT 1A or 2C agonists may change ISF Aβ levels, but any effects would be confounded by these off-target interactions. Identifying the minimum concentration that reduces ISF Aβ for each compound would be valuable from a therapeutic standpoint. However, the primary focus of this study was 5-HT signaling pathways instead of AD therapy.

5-HTR subtypes 4, 6 and 7 activate G_s_ proteins, typically increase cAMP levels, and induce PKA activation [15, 20]. The strong reduction of ISF Aβ induced by stimulating these receptors suggests that PKA may be involved. We showed that inhibition of PKA activity with two structurally-distinct, selective inhibitors significantly increased ISF Aβ and abolished the effects of SSRI (Fig. 3). This result suggests that PKA activity is necessary for the reduction in ISF Aβ by serotonergic signaling. Interestingly, the increase in Aβ after PKA inhibition was of a similar magnitude to the increase observed after ERK inhibition [12, 38]. The interactions between these two kinases are complex. Stimulating PKA with serotonin has been shown to induce ERK activation, but there is evidence that ERK can activate PKA after exposure to brain derived neurotrophic factor (BDNF) [20, 47]. However, treating APP/PS1 mice with BDNF had no effect on ISF Aβ [38]. Further work will be necessary to understand the relationship between these two kinases following SSRI treatment.

### Citalopram does not change expression of Aβ metabolism related genes after acute SSRI treatment

We have shown that ERK inhibition raises Aβ and abolishes the effects of citalopram [12]. Generally, ERK can act within two cellular compartments; ERK can translocate into the nucleus to modify gene expression or can phosphorylate proteins in the cytoplasm. We saw no significant changes in expression in the major genes related to Aβ metabolism, serotonin receptors, or signaling pathways 16 h after SSRI treatment (Fig. 4). However, these results do not necessarily exclude the role of gene expression in Aβ production after SSRI treatment; other studies have shown reductions in secretase-related genes after chronic SSRI administration [12, 45]. However, those reductions were seen after months of treatment whereas our data were obtained after only a few hours. More research is necessary to understand when expression of Aβ metabolism genes begins to decline after SSRI treatment and what impact this has on Aβ. The rapid reduction in ISF Aβ we observe in the acute timeframe suggests a post-translational response to alter enzymatic activity. Additionally, our qPCR data also suggest ERK is acting outside the nucleus to reduce Aβ.

### α-secretase mediates the effects of serotonin-induced reduction of Aβ

ADAM10 is the constitutive α-secretase protein in neurons, however ADAM17 and MMP9 can act as α-secretase as well [3, 41, 42]. Using a broad spectrum inhibitor of ADAM and MMP enzymes, GM6001, we showed that blocking α-secretase enzymatic activity alone increases ISF Aβ levels rapidly and, more importantly for this study, completely blocks the SSRI-dependent suppression in ISF Aβ levels (Fig. 5). Admittedly, broadly inhibiting ADAM/MMP proteins with GM6001 is mechanistically complicated; ADAM/MMP proteases could affect both Aβ generation and Aβ clearance [3, 48]. However, previous studies [12] have found citalopram SSRI does not affect Aβ clearance, meaning the effect here is likely on Aβ production. Unfortunately, the broad range of targets for GM6001 prevents us from identifying which α-secretase protease is responsible for the reduction in ISF Aβ after SSRI. Future studies using a more targeted approach with selective inhibitor compounds or genetic manipulation of the putative α-secretases will be necessary to answer that question.

Interestingly, treatment with a SSRI reduces both ISF Aβ levels and ISF sAPP-α levels. This result is actually opposite of what one might expect if serotonin increases α-secretase activity; however, the sAPP fragments have their own metabolic pathways that are not fully understood. The idea that APP cleavage produces a 1:1 relationship between all the fragments is not necessarily true. We find this new data intriguing and believe future studies are important and necessary to uncover the mechanisms are work here.

**Fig. 6:**
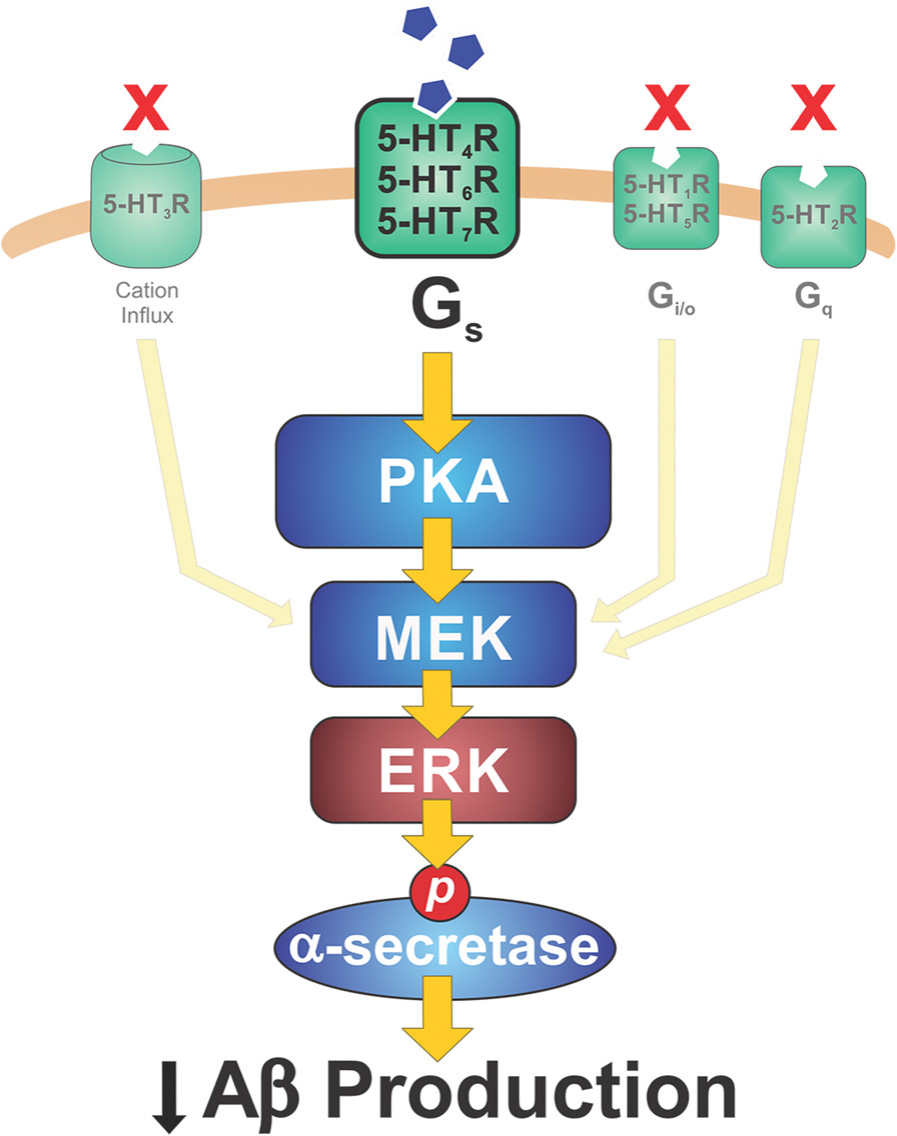
Model of Aβ reduction by serotonergic signaling. Serotonin binding to 5-HT_4_R, 5-HT_6_R, and 5-HT_7_R leads to the activation of PKA. PKA subsequently activates the MEK and ERK cascade. Activated ERK remains in the cytoplasm and phosphorylates α-secretase to increase its activity and reduce Aβ production

Our results suggest the simplified model presented in Fig. 6. Serotonin activates 5-HT_4_R, 5-HT_6_R, and 5-HT_7_R which then activate PKA. PKA signaling activates MEK and ERK. We hypothesize that activated ERK phosphorylates an α-secretase in the cytoplasm to increase its activity and reduce ISF Aβ. There is evidence that phosphorylation by ERK can increase α-secretase enzymatic activity [49]. ERK activation increases cleavage activity of ADAM17 in vitro, and mutating the putative ERK phosphorylation site blocks this increase [50]. Interestingly, ADAM10 contains a similar ERK consensus site which suggests it may also be an ERK substrate [51, 52]. Future research will determine if ERK directly phosphorylates α-secretase after SSRI exposure.

Activation of 5-HTR subtypes 4, 6, and 7 all reduce ISF Aβ by ~25 % which is the same amount as either SSRI treatment or direct 5-HT infusion (Fig. 1a, b). This suggests that these receptors likely activate a common signaling pathway, and activity of any of these receptors is sufficient to fully activate this pathway to suppress Aβ. Supportive of this hypothesis is that antagonizing one receptor alone does not change ISF Aβ, but antagonizing two receptors is necessary to increase Aβ by 30 % (Fig. 2). This suggests there is functional redundancy to serotonergic suppression of Aβ generation.

## Conclusions

The dramatic reductive effects of SSRIs on Aβ in mice and humans are encouraging for AD therapy. Millions of individuals world-wide use SSRIs on a daily basis for Depression and other disorders; however, these agents are not without side effects. 5-HTRs can be found throughout the digestive system [53, 54], and their stimulation by SSRI therapy can cause unpleasant digestive problems [55]. Also, stimulation of 5-HT_2_R in vascular tissue can lead to constriction of blood vessels and sexual side effects [55, 56]. Focusing AD therapy on a single or subset of 5-HTRs may reduce some of the side effects of SSRI treatment. In this report, we have shown activation of 5-HT_4_R, 5-HT_6_R, and 5-HT_7_R by selective agonists can significantly reduce ISF Aβ in mice by 25 %. In other mouse studies, a similar reduction of Aβ levels is sufficient to completely block plaque growth and significantly reduce new plaque formation [13, 57]. The safety and efficacy of selective agonists for these receptors need to be tested in humans; however, they offer a tantalizing path to treating AD.

## Methods

### Animals

All experiment protocols using animals were performed in accordance to the guidelines established by the Animal Studies Committee at Washington University. We bred *APPswe/PS1∆E9* hemizygous mice (Jackson Laboratory) [58] to wild type C3H/B6 mice and aged the *APP/PS1*^*+/-*^ offspring to 2–3.5 months for experiments. Mice were screened for APPswe and PS1ΔE9 transgenes by PCR from toe DNA.

### Compounds

All pharmaceutical compounds were ordered from Tocris Biosciences except for serotonin hydrochloride (Sigma-Aldrich). All compounds delivered by reverse microdialysis were diluted in microdialysis buffer consisting of artificial cerebrospinal fluid (aCSF) with 0.15-2 % BSA (Sigma-Aldrich). Specificity of each compound for its target was established by activity assays against libraries of receptors as described in previous literature [59–68]. The selectivity limit is the concentration above which the compound begins to stimulate off-target receptors. Concentrations used took into account the IC_50_ or K_i_ for each compound and only 10 % delivery across the membrane at a 1 μl/min flow rate (Table 1). This small volume of each compound would be diluted even further in brain CSF. Agonists are 5-175 times more selective for their targets than other receptor types. Citalopram hydrobromide (Toronto Research Chemicals) was diluted in PBS and injected i.p. at 10 mg/kg of body weight.

### In vivo Aβ microdialysis

In vivo microdialysis to measure brain ISF Aβ and sAPP-α in the hippocampus of freely moving APP/PS1 mice was performed similar to previously described [12, 24, 28]. This method captures soluble molecules in the extracellular fluid that are below the 30 kDa molecular weight cutoff of the probes. Under volatile isoflurane anesthetic, guide cannula (BR-style; Bioanalytical Systems or Atmos; EICOM) were cemented above the left hippocampus (3.1 mm behind Bregma, 2.5 mm lateral to midline, and 1.2 mm below dura at a 12° angle). Two millimeter microdialysis probes were inserted through the guides so their membranes were completely contained in the hippocampus (30 kDa BR-2; Bioanalytical Systems; 1,000 kDa AtmosLM; EICOM). Microdialysis buffer was aCSF (perfusion buffer in mM: 1.3 CaCl_2_, 1.2 MgSO_4_, 3 KCl, 0.4 KH_2_PO_4_, and 122 NaCl, pH 7.35) containing 0.15-2 % BSA (Sigma-Aldrich) that was filtered through a 0.1 μM membrane. The flow rate was 1.0 μL/min. Samples were collected every 60 or 90 min into a refrigerated fraction collector (Univentor Limited) in polypropylene tubes and assessed for Aβ_x-40_ by sandwich ELISA. Basal ISF Aβ levels were defined as the mean concentration of Aβ over the 9 h preceding drug administration. All Aβ values were normalized to the basal Aβ concentration for each animal. After establishing baseline ISF Aβ, pharmaceutical agents (serotonin, 5-HTR agonists, 5-HTR antagonists, enzyme inhibitors) were diluted in microdialysis perfusion buffer and infused directly into the hippocampus by reverse microdialysis (Table 1).

### Aβ sandwich ELISA

ISF Aβ_x-40_ levels were measured using sandwich ELISAs as described [12]. This ELISA detects both human and murine Aβ. A mouse anti-Aβ_40_ antibody (mHJ2; 10 μg/ml) against the C-terminus of Aβ was used to capture peptides and a biotinylated central domain antibody (mHJ5.1; 75 ng/ml) was used to detect them. This was followed by a streptavidin poly-HRP-40 assay to measure Aβ concentration (Fitzgerald Industries). All steps included washes with PBS containing 0.05 % Tween-20. The standard curve for the ELISA was synthetic Aβ_40_ (American Peptide) taken from a stock in formic acid to remove preformed aggregates. ELISA sample buffers included sufficient Tris to neutralize pH of the formic acid. To detect Aβ oligomers, ELISA plates were coated a mouse anti-Aβ antibody (mHJ3.4; 10 μg/ml) to capture of N-terminus of the peptide then detected using the same monoclonal antibody that was biotinylated (500 ng/ml). None of the buffers for this assay contain detergents as such agents can artificially produce Aβ aggregates. The standard curve for this ELISA is a synthetic dimer of human Aβ_40_ that contains a Ser26Cys mutation that enables us to covalently crosslink the peptides together. The dimer is then specifically isolated by size exclusion chromatography. Aβ dimers were a generous gift from Dr. David Brody, Washington University. The oligomer assay is similar to one used in [69]. All ELISAs were developed using Super Slow ELISA TMB (Sigma-Aldrich) and absorbance read on a Bio-Tek Epoch plate reader at 650 nm.

### Quantitative real-time PCR (qPCR)

2.5 month old APP/PS1 mice were given 10 mg/kg i.p. injections of citalopram or PBS. Mice were sacrificed 16 h later, and their hippocampi were microdissected. Total RNA were extracted using the RNeasy Mini kit (Qiagen) and reverse transcribed using the High Capacity cDNA Reverse Transcription kit (Life Technologies). Individual primers were designed using Harvard Medical School Primer Bank (http://pga.mgh.harvard.edu/primerbank/index.html) [70–72]. See Additional file 1 for primer sequences and database identification numbers. qPCR was performed using the Fast SYBR Green Master Mix (Applied Biosystems) in ABI 7900HT (Applied Biosystems) with the default thermal cycling program. Dissociation curves were analyzed following qPCR assay to confirm primer efficacy. Endogenous mouse GAPDH was used as a normalization reference. Relative mRNA levels were calculated by comparative Ct method using the ABI 7900HT Sequence Detection Systems version 2.0.5 and GenEx version 5 software (MultiD analyses). Levels of each mRNA were statistically compared to CaMKII mRNA levels, which our previous studies have shown does not mediate suppression of brain Aβ levels [38].

### SDS-PAGE/western blot

ISF samples collected from 1,000 kDa MWCO microdialysis probes (AtmosLM, Eicom) in APP/PS1 mice treated with citalopram for 16 h were utilized for Western blotting for soluble APP-α. Hippocampus from APP/PS1 mice treated with citalopram for 16 h was lysed in 1 % Triton X-100, 0.1 % SDS in PBS by sonication. 20 μg of total protein was loaded per lane on SDS-PAGE gels. SDS-PAGE was performed using 4–12 % Bis-Tris NuPAGE gels (Invitrogen, Carlsbad, CA) under reducing conditions with 24 μl of microdialysis sample loaded into each lane. Samples were loaded onto the gels in alternating basal and drug-treated samples. Nitrocellulose blots were probed with either mouse-anti-human APP antibody directed against the C-terminus of sAPP-α (m6E10; BioLegend), rabbit-anti-ADAM10 antibody (H-300; Santa Cruz Biotech), rabbit-anti-ADAM17 (Abcam), goat-anti-MMP2 (C-19; Santa Cruz Biotech), or mouse-anti-GAPDH (Sigma-Aldrich) followed by either a sheep-anti-mouse antibody (GE Life Sciences), donkey-anti-rabbit antibody (Santa Cruz Biotech), or a donkey-anti-goat antibody (Santa Cruz Biotech) conjugated to peroxidase. Bands were detected with Lumigen-TMA6 (Amersham, Piscataway, NJ) and captured digitally using the Kodak ImageStation 440CF. Densitometry was performed using the Kodak 1D Image Analysis software. Tissue protein levels were normalized to GAPDH for each lane, then normalized to % vehicle for each blot, followed by data from two blots being combined for analysis.

### Statistical analysis

Data in figures are presented as mean ± SEM. All statistical analysis was performed using Prism version 6.0 for Windows (GraphPad). For analysis of microdialysis data at endpoints, the final 3 data points for a given treatment phase were averaged, and one-way ANOVA analyses were performed with Dunnett’s corrections for multiple comparisons. Comparison of only two groups was performed using a two-tailed, unpaired Student *t*-test method. For qPCR analysis, mean levels of gene expression were analyzed by one-way ANOVA with Dunnett’s corrections for multiple comparisons. Values were accepted as significant if *p-*value ≤ 0.05.

## Additional file

Additional file 1: Supplemental Data and Primer Sequences. (DOCX 358 kb)
