# Supplementary material for: Redundant Gs-coupled serotonin receptors regulate amyloid-β metabolism in vivo
Source: Mol Neurodegener. 2016 Jun 18;11:45. doi: 10.1186/s13024-016-0112-5 (PMC4912779; doi:10.1186/s13024-016-0112-5)
Supplement: Additional file 1: — Supplemental Data and Primer Sequences. (DOCX 358 kb) [file 13024_2016_112_MOESM1_ESM.docx]

**
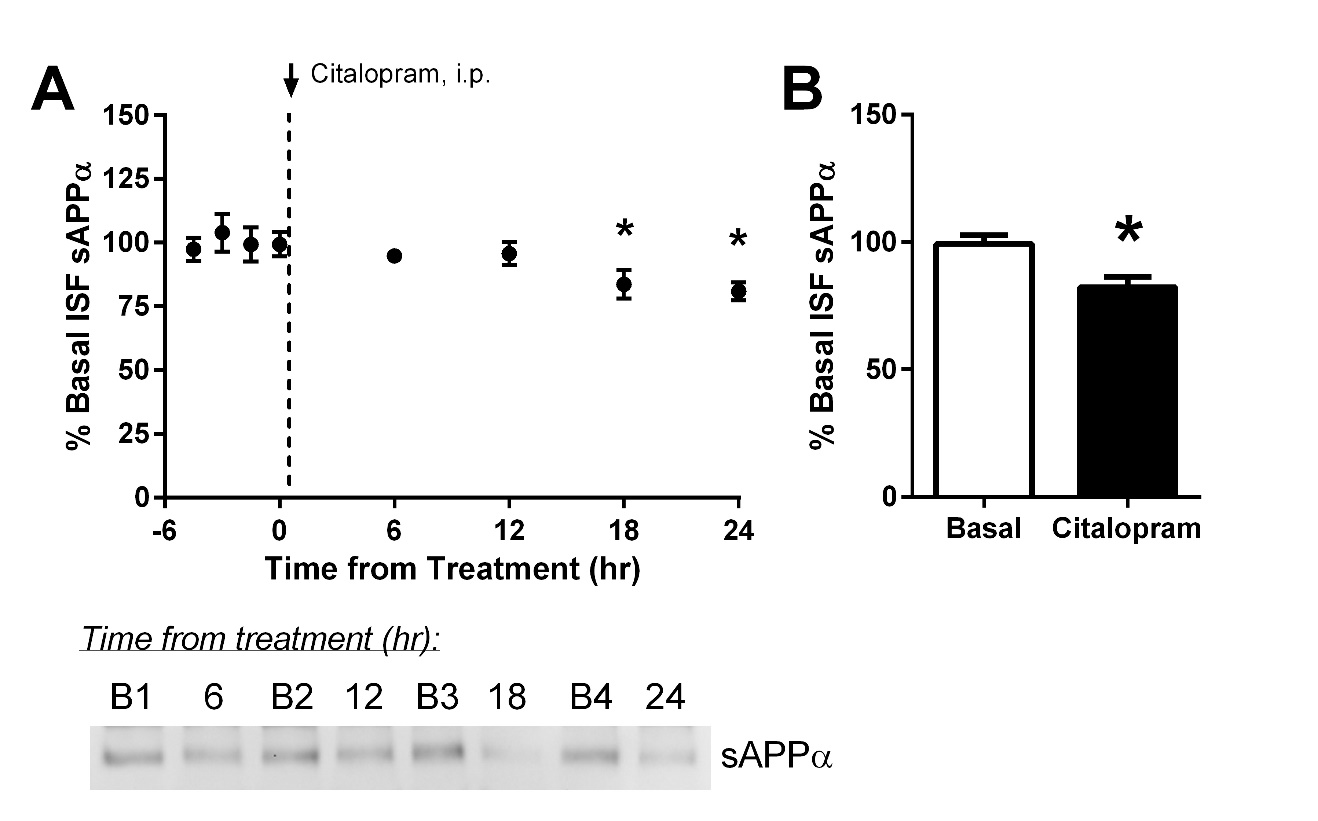
**

**Supplemental Figure S1: Effect of citalopram on ISF soluble APPα levels.** 2.5 month old APP/PS1 mice were implanted with 1,000 kDa MWCO microdialysis probes into the hippocampus in order to sample large proteins and molecules. Microdialysis samples were collected every 90 minutes through the entire study. **A.** Basal ISF sAPPα was measured over a 6 hour period followed by administration of citalopram (10mg/kg i.p.) with sampling for an additional 24 hours. Plot of normalized ISF sAPPα levels over time with representative Western blot (basal samples B1-4 and 6, 12, 18, and 24 hours after citalopram treatment). **B**. By 18-24 hour after citalopram administration, ISF sAPPα decreased by 17.4 ± 4.0% compared to basal levels in the 3 hours prior to treatment (p < 0.05; n = 4). Data presented as mean ± SEM.

**
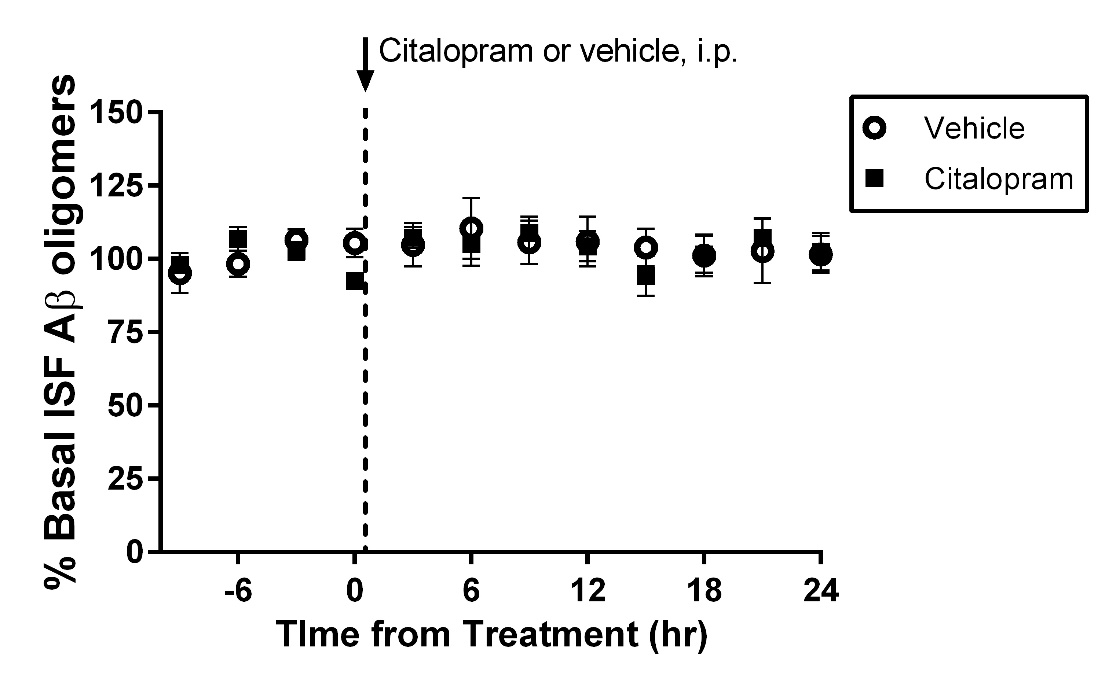
**

**Supplemental Figure S2: Effect of citalopram on ISF Aβ oligomers in young APP/PS1 mice.** 2.5 month old APP/PS1 mice were implanted with 1,000 kDa MWCO microdialysis probes into the hippocampus. ISF Aβ oligomer levels were measured at baseline and for 24 hours after citalopram (10mg/kg i.p.) or vehicle administration. There was no discernible effect of citalopram on Aβ oligomers (n = 6). Data presented as mean ± SEM.


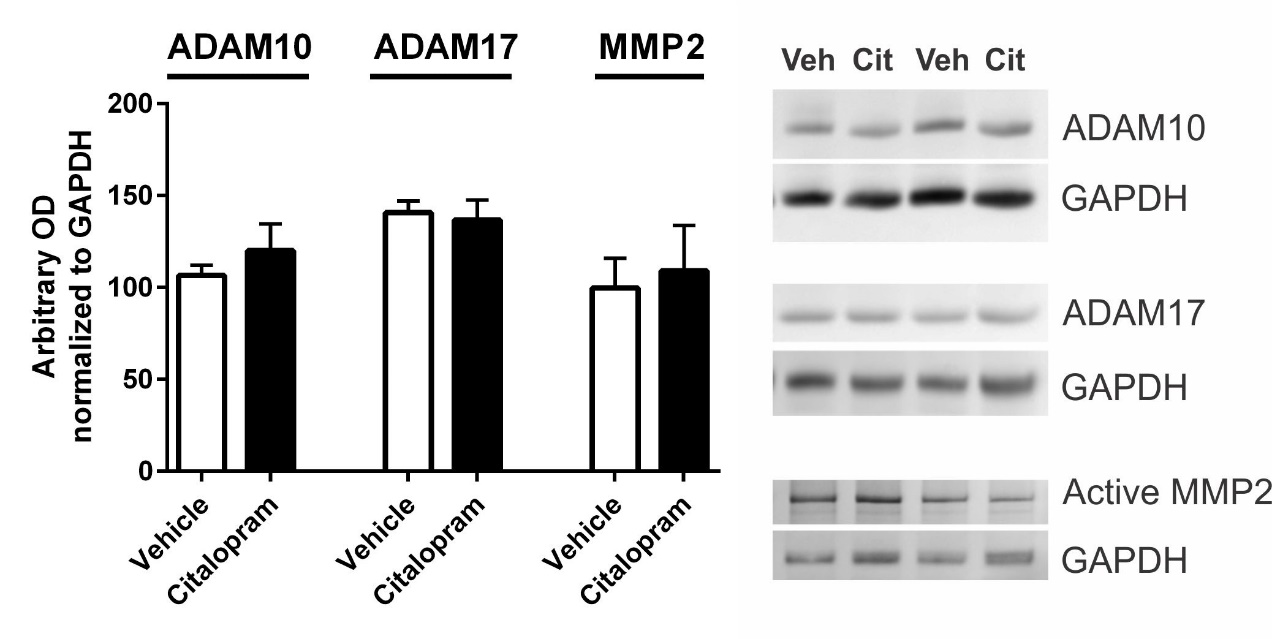


**Supplemental Figure S3: Westerns ADAM10, ADAM17, MMP2: Effect of citalopram on putative α-secretase protein expression.** 2.5 month old APP/PS1 mice were treated with vehicle or citalopram (10mg/kg ip) and sacrificed 16 hours to harvest hippocampus for biochemical analysis. Protein levels of ADAM10, ADAM17 and MMP2 were determined by Western blot with normalization to GAPDH. No significant differences in proteins levels were detected for any of the putative α-secretases (n=8 per group). Data presented as mean ± SEM with accompanying representative Western blot images for each protein.

| **Gene**  **Name** | **Sequence** | **Gene**  **Name** | **Sequence** | **Gene Name** | **Sequence** |
| --- | --- | --- | --- | --- | --- |
| ADAM10 | GTGCCAAACGAGCAGTCTCA | GAPDH | AGGTCGGTGTGAACGGATTTG | PKA Cβ | AGGGCAGGACATGGACATTG |
|  | ATTCGTAGGTTGAACTGTCTTCC |  | TGTAGACCATGTAGTTGAGGTCA |  | CGCCTTATTGTAACCCTTGCTG |
| ADAM17 | AGGACGTAATTGAGCGATTTTGG | JNK1 | AGCAGAAGCAAACGTGACAAC | PKC α | GTTTACCCGGCCAACGACT |
|  | TGTTATCTGCCAGAAACTTCCC |  | GCTGCACACACTATTCCTTGAG |  | GGGCGATGAATTTGTGGTCTT |
| Aph-1 | CCGCGCTCGCTCTTTATGT | JNK2 | TCAGTGGGTTGCATCATGGG | Presenilin 1 | GGTGGCTGTTTTATGTCCCAA |
|  | TGTACTGGTCCATCTCTGTTGT |  | GGATGGTGTTCCTAGCTGTTCA |  | CAACCACACCATTGTTGAGGA |
| APP | TGCAGAATTCCGACATGACT | JNK3 | CCATGTCTGTGTTCTTTCTCACG | Presenilin 2 | TGCCTGTCACGCTGTGTATG |
|  | GCCTTTGTTTGAACCCACAT |  | TTGGTTCCAACTGTGAAGAGTC |  | GTTAAGCACGGAGTTGAGGAG |
| β-arrestin 2 | GGCAAGCGCGACTTTGTAG | LRP1 | ACTATGGATGCCCCTAAAACTTG | PSEN2 | ATCTTGGTGGATTTGCGTTCC |
|  | GTGAGGGTCACGAACACTTTC |  | GCAATCTCTTTCACCGTCACA |  | GCGCCAAACATAGCCTTTGATTT |
| BACE1 | CAGTGGGACCACCAACCTTC | MEK1 | AAGGTGGGGGAACTGAAGGAT | 5-HT_1A_R | CATCGCGCTAGACAGGTACTG |
|  | GCTGCCTTGATGGACTTGAC |  | CGGATTGCGGGTTTGATCTC |  | CAATGAGCCAAGTGAGCGAGA |
| Basigin | GTGGCGTTGACATCGTTGG | MEK2 | GTTACCGGCACTCACTATCAAC | 5-HT_2A_R | TAATGCAATTAGGTGACGACTCG |
|  | CTATGTACTTCGTATGCAGGTCG |  | CCTCCAGCCGCTTCCTTTG |  | GCAGGAGAGGTTGGTTCTGTTT |
| B-Raf | AATTTGGTGGAGAGCATAACCC | MMP2 | CCTGGACCCTGAAACCGTG | 5-HT_3A_R | TGTGTACGTGCATCATCGAGG |
|  | ACGGTGTCCATTGATGCAGAG |  | TCCCCATCATGGATTCGAGAA |  | GCACATCAAAGGGGAAGTTGTAG |
| CaMKII | TGGAGACTTTGAGTCCTACACG | MMP9 | GGACCCGAAGCGGACATTG | 5-HT_4_R | AGTTCCAACGAGGGTTTCAGG |
|  | CCGGGACCACAGGTTTTCA |  | GAAGGGATACCCGTCTCCGT |  | CAGCAGGTTGCCCAAGATG |
| cFOS | CGGGTTTCAACGCCGACTA | Neprilysin | CTCTCTGTGCTTGTCTTGCTC | 5-HT_5B_R | TTGCTGATCGCTGCCACTTT |
|  | TTGGCACTAGAGACGGACAGA |  | GACGTTGCGTTTCAACCAGC |  | GTCGAGGCCACCAAGTTATGT |
| ERK1 | ACCACATTCTAGGTATCTTGGGT | Nicastrin | TCCGTGGTACTGGCAGGATT | 5-HT_6_R | GCATAGCTCAGGCCGTATGTG |
|  | AGTTTCGGGCCTTCATGTTAAT |  | CCCCTGTATCCCCACTAATTGA |  | CGCATGAAGAGGGGATAGATGA |
| ERK2 | GGTTGTTCCCAAATGCTGACT | PKA Cα | AGATCGTCCTGACCTTTGAGT | 5-HT_7_R | CCTTACCTCCTCTCTTCGGATG |
|  | CAACTTCAATCCTCTTGTGAGGG |  | GGCAAAACCGAAGTCTGTCAC |  | TGGAGTAGATCGTGTAGCCAAA |

**Additional Table 1: Primer sequences for qPCR analysis.** Nucleotide sequences for each primer are listed here. The forward primer is listed first in each sequence pair. Abbreviations: a disintegrin and metallopeptidase domain (ADAM), anterior pharynx defective 1 (Aph-1), amyloid precursor protein (APP), beta-site APP-cleaving enzyme (BACE1), calcium-calmodulin dependent kinase II (CaMKII), FBJ osteosarcoma oncogene (cFOS), extracellular signal-regulated kinase (ERK), glyceraldehyde 3-phosphate dehydrogenase (GAPDH), c-Jun N-terminal kinase (JNK), low density lipoprotein receptor-related protein 1 (LRP1), MAP kinase-ERK kinase (MEK), matrix metallopeptidase (MMP), protein kinase A catalytic unit alpha (PKA Cα), protein kinase A catalytic unit beta (PKA Cβ), protein kinase C alpha (PKC α), presenilin enhancer 2 (PSEN2), serotonin receptor (5-HTR).

| **Gene**  **Name** | **PrimerBank**  **ID Number** | **Gene**  **Name** | **PrimerBank**  **ID Number** | **Gene Name** | **PrimerBank**  **ID Number** | **Gene Name** | **PrimerBank**  **ID Number** |
| --- | --- | --- | --- | --- | --- | --- | --- |
| ADAM10 | 150378457c2 | cFOS | 6753894a1 | MEK2 | 31560267a1 | Presenilin 2 |  |
| ADAM17 | 34328548a1 | ERK1 | 93102422c3 | MMP2 | 47271505c2 | PSEN2 |  |
| Aph-1 | 146198522c1 | ERK2 | 33090821a1 | MMP9 |  | 5-HT_1A_R | 162135953c3 |
| APP |  | GAPDH | 6679937a1 | Neprilysin | 31543255a1 | 5-HT_2A_R | 27753985a1 |
| BACE1 | 31981412a1 | JNK1 | 7710060a1 | Nicastrin | 31981205a1 | 5-HT_3A_R | 153791844c2 |
| Basigin | 2808468a1 | JNK2 | 26327765a1 | PKA Cα | 7110693a1 | 5-HT_4_R | 6680325a1 |
| B-Raf | 7271247a1 | JNK3 | 28892799a1 | PKA Cβ | 255958317c3 | 5-HT_5B_R | 6754260a1 |
| β-arrestin 2 | 21703856a1 | LRP1 | 6678720a1 | PKC α | 6755078a1 | 5-HT_6_R | 118130478c3 |
| CaMKII | 161086916c1 | MEK1 | 6678794a1 | Presenilin 1 | 6679493a1 | 5-HT_7_R | 113865997c3 |

**Additional Table 2: Primer identification numbers for qPCR analysis.** The majority of primers were designed by the Harvard Primer bank. The identification numbers allow for rapid searching and cataloging. Primer sets left blank were designed by hand. Abbreviations: a disintegrin and metallopeptidase domain (ADAM), anterior pharynx defective 1 (Aph-1), amyloid precursor protein (APP), beta-site APP-cleaving enzyme (BACE1), calcium-calmodulin dependent kinase II (CaMKII), FBJ osteosarcoma oncogene (cFOS), extracellular signal-regulated kinase (ERK), glyceraldehyde 3-phosphate dehydrogenase (GAPDH), c-Jun N-terminal kinase (JNK), low density lipoprotein receptor-related protein 1 (LRP1), MAP kinase-ERK kinase (MEK), matrix metallopeptidase (MMP), protein kinase A catalytic unit alpha (PKA Cα), protein kinase A catalytic unit beta (PKA Cβ), protein kinase C alpha (PKC α), presenilin enhancer 2 (PSEN2), serotonin receptor (5-HTR).
